# Detection of Clade 2.3.4.4b Avian Influenza A(H5N8) Virus in Cambodia, 2021

**DOI:** 10.3201/eid2901.220934

**Published:** 2023-01

**Authors:** Kimberly M. Edwards, Jurre Y. Siegers, Xiaoman Wei, Ammar Aziz, Yi-Mo Deng, Sokhoun Yann, Chan Bun, Seng Bunnary, Leonard Izzard, Makara Hak, Peter Thielen, Sothyra Tum, Frank Wong, Nicola S. Lewis, Joe James, Filip Claes, Ian G. Barr, Vijaykrishna Dhanasekaran, Erik A Karlsson

**Affiliations:** University of Hong Kong, Hong Kong, China (K.M. Edwards, X. Wei, V. Dhanasekaran);; Institut Pasteur du Cambodge, Phnom Penh, Cambodia (J.Y. Siegers, S. Yann, E.A. Karlsson);; Peter Doherty Institute for Infection and Immunity, Melbourne, Victoria, Australia (A. Aziz, Y.-M. Deng, I.G. Barr);; National Animal Health and Production Research Institute, Phnom Penh (C. Bun, S. Bunnary, S. Tum);; Australian Center for Disease Preparedness, Geelong, Victoria, Australia (L. Izzard, F. Wong);; Food and Agriculture Organization of the United Nations, Phnom Penh (M. Hak);; Johns Hopkins University, Baltimore, Maryland, USA (P. Theilen);; Royal Veterinary College, London, UK (N.S. Lewis, J. James);; OIE/FAO International Reference Laboratory for Avian Influenza, Swine influenza and Newcastle Disease, Weybridge, UK (N.S. Lewis);; Food and Agriculture Organization of the United Nations, Bangkok, Thailand (F. Claes)

**Keywords:** avian influenza virus, pathogenic, H5N8, influenza, respiratory infections, viruses, zoonoses, food safety, live poultry markets, disease surveillance, whole-genome sequencing, phylogenetics, Cambodia

## Abstract

In late 2021, highly pathogenic avian influenza A(H5N8) clade 2.3.4.4b viruses were detected in domestic ducks in poultry markets in Cambodia. Surveillance, biosafety, and biosecurity efforts should be bolstered along the poultry value chain to limit spread and infection risk at the animal–human interface.

Since 2014, highly pathogenic avian influenza viruses (HPAIVs) with H5 hemagglutinin (HA) genes grouped in the genetic clade 2.3.4.4 have spread globally causing severe outbreaks in Africa, Europe, Asia, and most recently, North America (*1*). These viruses cause devastating outbreaks in poultry, rapidly evolve, and continuously reassort with other avian influenza viruses (AIVs), posing a threat to food security in many parts of the world and substantial zoonotic infection risk.

## The Study

Since 2017, the Institut Pasteur du Cambodge and National Animal Health and Production Research Institute in Cambodia have partnered with the Food and Agriculture Organization of the United Nations to enhance ongoing longitudinal AIV surveillance in live bird markets and poultry storage facilities throughout Cambodia. This active surveillance reveals high levels of AIV circulation with ≈30%–50% of ducks and ≈20%–40% of chickens testing positive for various influenza A subtypes. Most detected HPAIVs were H5N1 HA clade 1 viruses during 2005–2014 and H5N1 HA clade 2.3.2.1c viruses since 2014; H5N6 clades 2.3.4.4g and 2.3.4.4h were detected sporadically during 2018–2020 (Appendix 1, Table 1). Other subtypes also circulate, including novel H7Nx low pathogenicity avian influenza viruses (LPAIVs) (*2*,*3*).

**Figure 1:**
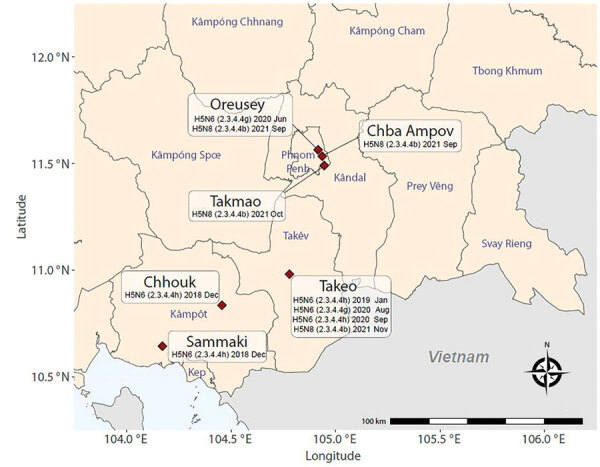
Location of live bird markets where highly pathogenic clade 2.3.4.4b avian influenza A(H5N8) viruses were detected in Cambodia during 2018–2021. The map shows where both H5N6 and H5N8 subtypes of avian influenza A were detected.

During active surveillance of live bird markets (National Ethics Committee for Health Research Approval no. 149/NECHR/2020) in late 2021, domestic ducks (*Anas platyrhynchos*) at Orussey (Phnom Penh, n = 1), Takmao (Kandal, n = 2), Chba Ampov (Phnom Penh, n = 1), and Takeo (Takeo, n = 1) tested positive for HPAIV H5 HA but negative for neuraminidase (NA) subtype N1 by real time reverse transcription PCR (RT-PCR). We determined these samples were the H5N8 subtype after further RT-PCR analysis (Appendix 1). Positive samples originated from Orussey and Chba Ampov markets during week 37, Takmao market during week 41, and Takeo market during week 46 of 2021 (Figure 1). Full genome sequencing on a GridION instrument (Oxford Nanopore Technologies, https://www.nanoporetech.com) confirmed these samples were HPAIV H5N8 and H5 HA clade 2.3.4.4b (*4*).

All H5N8 HA sequences from Cambodia encoded proteins with 2-4 amino acid differences relative to the clade 2.3.4.4b candidate vaccine strain A/Astrakhan/3212/2020(H5N8) (Table 1). HA mutations T192I and H276N (according to the H3 numbering system) were shared across all H5N8 HA proteins, whereas A188V occurred in 2 sequences, and E273K, T312S, and I339K each occurred only once. Those mutations did not correlate with previously reported phenotypic traits. Consistent with other clade 2.3.4.4b HA proteins, H5N8 viruses from Cambodia retained the HPAIV cleavage site motif, REKRRKR|GLF. The NA sequences did not contain stalk deletions or markers of antiviral drug resistance. However, other genes encoded amino acid residues associated with increased replication capacity and mammalian pathogenicity, including V89, V292, D309, R389, and T598 in PB2; G622 in PB1; D30, M43, and A215 in M1; and S42 and M106 in NS proteins (*5*).

**Table 1:** Amino acid mutations in hemagglutinin relative to the reference strain A/Astrakhan/3212/2020 in clade 2.3.4.4b avian influenza A(H5N8) viruses detected in Cambodia, 2021*

H5 clade 2.3.4.4b strain	HA amino acid position†					
	188	192	273	276	312	339
A/Astrakhan/3212/2020 (CVV)‡	A	T	E	H	N	I
A/duck/Cambodia/f6T241D4/2021		I	K	N	S	
A/duck/Cambodia/f4K241D3_C/2021	V	I		N		
A/duck/Cambodia/f4K241D4/2021	V	I		N		
A/duck/Cambodia/f1PPOreu241D3_C/2021		I		N		K
A/duck/Cambodia/f1PPChba241D6/2021		I		N		

*Blank cells indicate no mutation. HA, hemagglutinin. †Amino acids were numbered according to the hemagglutinin H3 numbering system.  ‡Candidate vaccine virus reference strain (GISAID accession no. EPI_ISL_1038924; https://www.gisaid.org).

We performed hemagglutination inhibition assays to assess potential cross-reactivity among the 2.3.4.4b viruses isolated from ducks in Cambodia by using 2 key reference viruses: A/Astrakhan/3212/2020, the recommended candidate vaccine virus for clade 2.3.4.4.b; and A/domestic_duck/England/074477/2021, a recently identified clade 2.3.4.4b virus from poultry associated with human infection in the United Kingdom (*6*). H5N8 viruses from Cambodia with V188, I192 and N276 in HA showed good recognition by antiserum raised against A/Astrakhan/3212/2020. However, A/duck/Cambodia/f1PPOreu241D3/2021 (with I192 and N276, and K339 in HA) and A/duck/Cambodia/f6T241D4/2021 (with I192, K273, N276, S312 in HA) showed reduced recognition by the antiserum (Table 2).

**Table 2:** Hemagglutination inhibition titers of isolated virus strains in study of clade 2.3.4.4b avian influenza A(H5N8) virus detected in Cambodia, 2021*

Strain	Titer
A/duck/Cambodia/f1PPOreu241D3/2021	40
A/duck/Cambodia/f4K241D4/2021	80
A/duck/Cambodia/f4K241D3/2021	80
A/duck/Cambodia/f6T241D4/2021	40
A/domestic_duck/England/074477/2021	80
A/Astrakhan/3212/2020/2020 (CVV)†	160

*Hemagglutination inhibition titer using ferret antiserum against the reference virus strain A/Astrakhan/3212/2020. †Candidate vaccine virus reference strain (GISAID accession no. EPI_ISL_1038924; https://www.gisaid.org).

**Figure 2:**
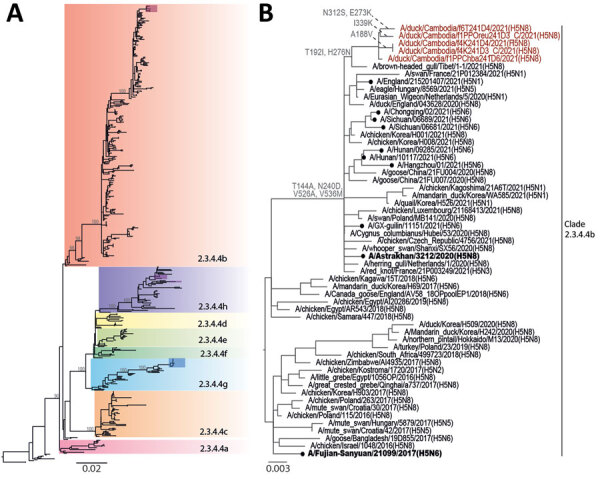
Phylogenetic analysis of the hemagglutinin genes of clade 2.3.4.4 avian influenza A(H5N8) viruses detected in Cambodia. Whole-genome sequencing of isolated viruses was performed and phylogenies were constructed using the maximum-likelihood method. A) Subclades of H5Nx clade 2.3.4.4. Recent isolates from Cambodia are shown in red, purple, and blue shaded boxes. B) Phylogeny of avian influenza A(H5N8) clade 2.3.4.4b isolates. Recent isolates from Cambodia are in red font and amino acid mutations are indicated at select nodes. Candidate vaccine viruses used as reference viruses are in bold font. Closed circles indicate cases of human infection with avian H5Nx viruses. Scale bars indicate nucleotide substitutions per site.

The H5N8 viruses from Cambodia shared >95.7% nucleotide sequence homology across their genomes and formed distinct monophyletic lineages in maximum-likelihood phylogenies of several genes (bootstrap support was 100%, except for the matrix protein gene, which was 89%; Appendix Figure 1), implying circulation of a single virus strain >10 weeks from September to November 2021. The H5 HA gene was likely derived from H5N8 viruses that have caused widespread outbreaks in poultry and wild birds across Eurasia since early 2020 (*7*) (Figure 2). N8 NA gene segments were closest to that of HPAIV H5N8 detected in wild and domestic waterfowl in China and Korea during 2020–21, sharing most recent ancestry with NA of A/*Cygnus_columbianus*/Hubei/116/2020(H5N8) that was collected in November 2020 (Appendix 1 Figure 1). Both the HA and MP gene segments were most closely related to A/brown-headed gull/Tibet/1–1/2021(H5N8), collected in May 2021 (Figure 2, panel B; Appendix 1 Figure 1). In contrast, the other gene segments encoding internal virus proteins were derived from LPAIV (Appendix 1 Figure 1). PB2 and PB1 genes shared common ancestry with LPAIV detected in ducks in Vietnam in 2020. PA and NP genes shared recent common ancestry with LPAIV isolated in 2019 from wild ducks in Korea (PA gene) and China (NP gene). The NS protein gene was most similar to that of LPAIV from ducks in China in 2018. Overall, HPAIV H5Nx clade 2.3.4.4b showed evidence of extensive genetic reassortment with LPAIV found in wild waterfowl, which frequently spillover to and from domestic poultry.

In addition to various LPAIVs, multiple H5 subtypes and clades circulate in Cambodia (Appendix 1 Table 1). H5N1 clade 2.3.2.1c viruses are detected regularly. H5N6 clade 2.3.4.4g viruses were found in Takeo and Orussey markets in chickens in 2019 and ducks in 2020, and H5N6 clade 2.3.4.4h viruses were detected sporadically in Kampot province in late 2018, Takeo province during 2019–2020, and Phnom Penh in 2020 (Figures 1, 2; Appendix 1 Figures 2–5). Therefore, detection of H5N8 clade 2.3.4.4b viruses in these same markets is a major concern because further reassortment might occur. Since 2018, outbreaks of reassorted HPAIV H5Nx clade 2.3.4.4b with NA subtypes N8, N6, N1, N3, and N5 have increased in frequency (*8*). These viruses have disseminated intercontinentally across migratory flyways and regionally via poultry trade, often causing considerable economic losses. In 2021, H5Nx clade 2.3.4.4b viruses caused severe outbreaks in Europe, Africa, and Asia, particularly in wild birds in western China and in domestic poultry in Vietnam (*9*). Since January 2022, HPAIV H5N1 clade 2.3.4.4b has been detected in waterfowl, birds of prey, and poultry across North America (*10*).

H5Nx clade 2.3.4.4b viruses also pose a zoonotic risk to humans and other species. In February 2021, a total of 7 cases of asymptomatic human infections with HPAIV H5N8 clade 2.3.4.4b were reported in poultry farm workers in Russia following a poultry outbreak (*11*). H5N6 clade 2.3.4.4 viruses have caused 79 human infection cases (including 33 cases in 2021) in China with ≈32 deaths since 2014 and 1 case in Laos (*12*), and 3 cases of H5Nx were reported in Nigeria (*9*). HPAIV H5Nx clade 2.3.4.4 viruses have also been detected in domestic cats in China and Korea (*1*) and red foxes in The Netherlands (*1*), and serologic evidence exists for infection in swine (*13*). More recently, HPAIV H5N1 clade 2.3.4.4b containing HA genes closely related to A/Astrakhan/3212/2020 have caused human infections in the United Kingdom (*14*) and United States (*15*). HPAIV H5N1 clade 2.3.3.4b has not been detected in Cambodia.

## Conclusions

Because of the global spread, economic impact, and zoonotic potential of HPAIV clade 2.3.4.4b viruses, active, longitudinal surveillance in live bird markets must be maintained in Cambodia, the Greater Mekong Subregion, and globally to monitor further introduction and reassortment events. In addition, surveillance of influenza-like illness needs to be maintained among persons in close contact with infected or deceased poultry. To combat the spread of HPAIV in Cambodia and other countries, viral monitoring, biosafety, and biosecurity efforts should be bolstered along the poultry value chain. Early warning and rapid control will limit infections at the animal–human interface to reduce potential pandemic risk.

Appendix 1 Additional information for detection of clade 2.3.4.4b avian influenza A(H5N8) virus in Cambodia, 2021.

Appendix 2 Authors and originating and submitting laboratories of the sequences from the GISAID database
