# Supplementary material for: Detection of Clade 2.3.4.4b Avian Influenza A(H5N8) Virus in Cambodia, 2021
Source: Emerg Infect Dis. 2023 Jan;29(1):170–4. doi: 10.3201/eid2901.220934 (PMC9796211; doi:10.3201/eid2901.220934)
Supplement: Appendix 1 — Additional information for detection of clade 2.3.4.4b avian influenza A(H5N8) virus in Cambodia, 2021. [file 22-0934-Techapp-s1.pdf]

Article DOI: https://doi.org/10.3201/eid2901.220934

Detection of Clade 2.3.4.4b Avian Influenza A(H5N8) Virus in Cambodia, 2021

Appendix 1

Materials and Methods

Sample Collection and Screening

We collected tracheal, cloacal, and environmental samples (August 2017–December 2021) from ducks and chickens as described previously (*1*). We extracted viral RNA by using the QIAamp Viral RNA Mini Kit (QIAGEN, https://www.qiagen.com) in accordance with the manufacturer's protocol. We screened all samples by real-time reverse transcription PCR (RT-PCR) of the matrix protein gene and then by using H5 HA-specific primers, as previously described (*2*). We propagated matrix gene-positive samples, if there was sufficient sample and high virus concentration (RT-PCR Ct<30), in 10-day-old embryonated chicken eggs for virus isolation. We tested original isolates for all known subtypes of avian influenza virus by using influenza multiplex RT-PCR.

Genome Sequencing

We amplified whole genomes of influenza A viruses from Cambodia by using custom Uni12/Inf-1 and Uni13/Inf-1 barcoded primers (*3*), kindly provided by Peter Thielen, and SSIII One-step RT-PCR with Platinum Taq High Fidelity kit (Thermo Fisher Scientific, https://www.thermofisher.com). We prepared sequencing libraries by using a ligation sequencing kit (Oxford Nanopore Technologies, https://www.nanoporetech.com). Samples were sequenced by using the GridION platform (Oxford Nanopore Technologies). We then demultiplexed, quality trimmed, and filtered sequencing reads by using Porechop software (https://www.github.com/rrwick/Porechop). We deposited a total of 159 gene segment sequences obtained from 20 influenza A viruses in GenBank under accession numbers ON716288–446.

To generate the consensus sequence, IRMA (Integrated Resource Management Applications, https://irma.nps.gov/Portal) was run with default settings. We manually inspected

consensus sequences for errors, such as insertion-deletion mutations and mixed bases, and corrected if required. We used a minimum 100-bp cutoff for depth coverage for all gene segments. The average per sample depth was 46,800 reads (minimum, 6,000; maximum, 150,000; median, 30,000) and the average per gene depth was 38,000 reads (minimum, 13,000; maximum, 86,000; median, 39,000). Several (4 out of 20) randomly selected viruses underwent resequencing by the World Health Organization Collaboration Center in Melbourne, Australia, for confirmation and quality control. Sequences that were problematic at the multibasic cleavage site were confirmed by using Sanger sequencing at Macrogen (https://www.macrogen.com). A positive control (A/H3N2) and several negative controls were included at each step of the sequencing protocol.

Phylogenetic Analysis

We used metadata from Nextstrain (*4*) (accessed on 2022 Jan 19) to prepare subsets of HPAIV H5 HA sequences, which were downloaded from the GISAID database (*5*) (https://www.gisaid.org, accessed on 2022 Jan 19). We downloaded all available N6 and N8 subtype neuraminidase sequences and genes encoding internal proteins of all H5Nx subtype viruses from GISAID and GenBank. For each of the internal gene datasets, we added the top 10 BLAST matches from GISAID and GenBank that were closest to the H5Nx sequences from Cambodia. We excluded duplicate (according to strain name), laboratory-derived, mixed subtype, and low coverage (<90% of full length) sequences from downstream analysis. We aligned sequences with MAFFT v.7.490 (*6*) and constructed phylogenetic trees by using the best-fit nucleotide substitution model in IQ-TREE v.2.1.4 (*7*). We visualized and annotated the trees by using FigTree v.1.4.4 (http://tree.bio.ed.ac.uk/software/figtree).

Hemagglutinin Inhibition Assays

We conducted hemagglutinin inhibition (HI) assays according to standard methods (*1*). We treated serum samples with a receptor-destroying enzyme and conducted erythrocyte adsorption before HI testing. In brief, 1 volume of heat-treated serum sample was added to 4 volumes of receptor-destroying enzyme (APHA Scientific, http://apha.defra.gov.uk/apha-scientific/index.htm). We incubated the resulting solution for 16 h at 37°C before heat-inactivation at 56°C for 1 h. We added packed erythrocytes from specific pathogen-free chickens at a ratio of 1:5 (erythrocyte:serum), incubated at room temperature for 30 min, centrifuged at 1000 ×*g*, and transferred the supernatant to a clean tube. We performed the HI assay at 4°C by

using 4 hemagglutinating units of the indicated H5 HPAIV strains as antigens, ferret antiserum against the reference virus, and chicken erythrocytes. We serially diluted serum samples 2-fold for HI assays starting at a dilution of 1:4 and tested the samples in duplicate. We included control wells for each sample to test for nonspecific hemagglutination, and positive antiserum and negative control serum (taken from specific pathogen-free chickens) were also tested for each assay.

Data Availability

Code and accession numbers are available at https://github.com/vjlab/cambodia-H5N8.

References

1. Suttie A, Tok S, Yann S, Keo P, Horm SV, Roe M, et al. Diversity of A(H5N1) clade 2.3.2.1c avian influenza viruses with evidence of reassortment in Cambodia, 2014–2016. PLoS One. 2019;14:e0226108. PubMed https://doi.org/10.1371/journal.pone.0226108

2. Siegers JY, Dhanasekaran V, Xie R, Deng Y-M, Patel S, Ieng V, et al. Genetic and antigenic characterization of an influenza A(H3N2) outbreak in Cambodia and the Greater Mekong Subregion during the COVID-19 pandemic, 2020. J Virol. 2021;95:e0126721. PubMed https://doi.org/10.1128/JVI.01267-21

3. Zhou B, Donnelly ME, Scholes DT, St George K, Hatta M, Kawaoka Y, et al. Single-reaction genomic amplification accelerates sequencing and vaccine production for classical and swine origin human influenza a viruses. J Virol. 2009;83:10309–13. PubMed https://doi.org/10.1128/JVI.01109-09

4. Hadfield J, Megill C, Bell SM, Huddleston J, Potter B, Callender C, et al. Nextstrain: real-time tracking of pathogen evolution. Bioinformatics. 2018;34:4121–3. PubMed https://doi.org/10.1093/bioinformatics/bty407

5. Shu Y, McCauley J. GISAID: global initiative on sharing all influenza data—from vision to reality. Euro Surveill. 2017;22:30494. PubMed https://doi.org/10.2807/1560-7917.ES.2017.22.13.30494

6. Katoh K, Misawa K, Kuma K, Miyata T. MAFFT: a novel method for rapid multiple sequence alignment based on fast Fourier transform. Nucleic Acids Res. 2002;30:3059–66. PubMed https://doi.org/10.1093/nar/gkf436

7. Nguyen L-T, Schmidt HA, von Haeseler A, Minh BQ. IQ-TREE: a fast and effective stochastic algorithm for estimating maximum-likelihood phylogenies. Mol Biol Evol. 2015;32:268–74. PubMed https://doi.org/10.1093/molbev/msu300

Appendix Table 1. Influenza A virus H5Nx subtypes isolated from live bird markets and sequenced in study of clade 2.3.4.4b avian influenza A(H5N8) virus detected in Cambodia, 2017–2021*

Subtype	H5Nx viruses detected					
(H5 HA clade)	2017	2018	2019	2020	2021	Total
H5N1 (2.3.2.1c)	12	19	33	9	12	85
H5N2 (2.3.2.1c)	0	1	1	0	0	2
H5N6 (2.3.4.4h)	0	3	1	1	0	5
H5N6 (2.3.4.4g)	0	0	0	10	0	10
H5N8 (2.3.4.4b)	0	0	0	0	5	5
Totals	12	23	35	20	17	110

*H, hemagglutinin; HA, hemagglutinin; N, neuraminidase.

Appendix Table 2. Amino acid mutations in HA from avian influenza A(H5N6) clade 2.3.4.4g viruses detected in Cambodia, 2021*

	HA amino acid position†												
H5 clade 2.3.4.4g strain name	5	94	140	151	165	167	192	199	242	276	371	379	507
A/chicken/Vietnam/Raho4-Cd-20–421/2020‡	V	P	Q	V	E	S	T	T	A	R	K	D	S
A/duck/Cambodia/e5PPOreu241D3/2020	I			L	K	N	E	A	E	N	R	N	T
A/duck/Cambodia/e5PPOreu241D4/2020	I	A		L	K	N	E	A	E	N	R	N	T
A/duck/Cambodia/e5PPOreu241D8/2020	I			L	K	N	E	A	E	N	R	N	T
A/duck/Cambodia/e8T241D6/2020	I		L	L	K	N	E	A	E	N	R	N	T
A/duck/Cambodia/e8T241D5/2020	I		L	L	K	N	E	A	E	N	R	N	T
A/duck/Cambodia/e8T241D9/2020	I		L	L	K	N	E	A	E	N	R	N	T
A/duck/Cambodia/e8T241D10/2020	I		L	L	K	N	E	A	E	N	R	N	T
A/duck/Cambodia/e8T241D15/2020	I		L	L	K	N	E	A	E	N	R	N	T
A/duck/Cambodia/e8T241D16/2020	I		L	L	K	N	E	A	E	N	R	N	T
A/duck/Cambodia/e8T241D11/2020	I		L	L	K	N	E	A	E	N	R	N	T

*Blank cells indicate no mutation. HA, hemagglutinin.
†H5N6 HA segments were compared to the reference strain A/chicken/Vietnam/Raho4-Cd-20–421/2020. Amino acids were numbered according to the hemagglutinin H3 numbering system.
‡Candidate vaccine virus (GISAID accession: EPI_ISL_1379443).

Appendix Table 3. Amino acid mutations in HA from avian influenza A(H5N6) clade 2.3.4.4h viruses detected in Cambodia, 2021*

	HA amino acid position†																
H5 clade 2.3.4.4h strain	4	5	10	41	60	92	125	142	166	187	192	202	214	222	242	450	536
A/Guangdong/18SF020/2018‡	V	V	S	D	V	N	R	Q	M	A	T	V	V	Q	A	K	M
A/duck/Cambodia/c18MKAP189/2018	M				I			T	K		A		M			R	V
A/duck/Cambodia/c18MKAP211/2018	M				I			T	K		A		M			R	V
A/duck/Cambodia/c18MKAP214/2018	M				I			T	K		A		M			R	V
A/chicken/Cambodia/c9T241C17T/2019	M				I	S		T	K		A	I					V
A/duck/Cambodia/e10T241C18/2020		F	N	N			S			V				R	T		V

*Blank cells indicate no mutation. HA, hemagglutinin.
†H5N6 HA segments were compared to the reference strain A/Guangdong/18SF020/2018. Amino acids were numbered according to the hemagglutinin H3 numbering system.
‡Candidate vaccine virus (GISAID accession no. EPI_ISL_337274).

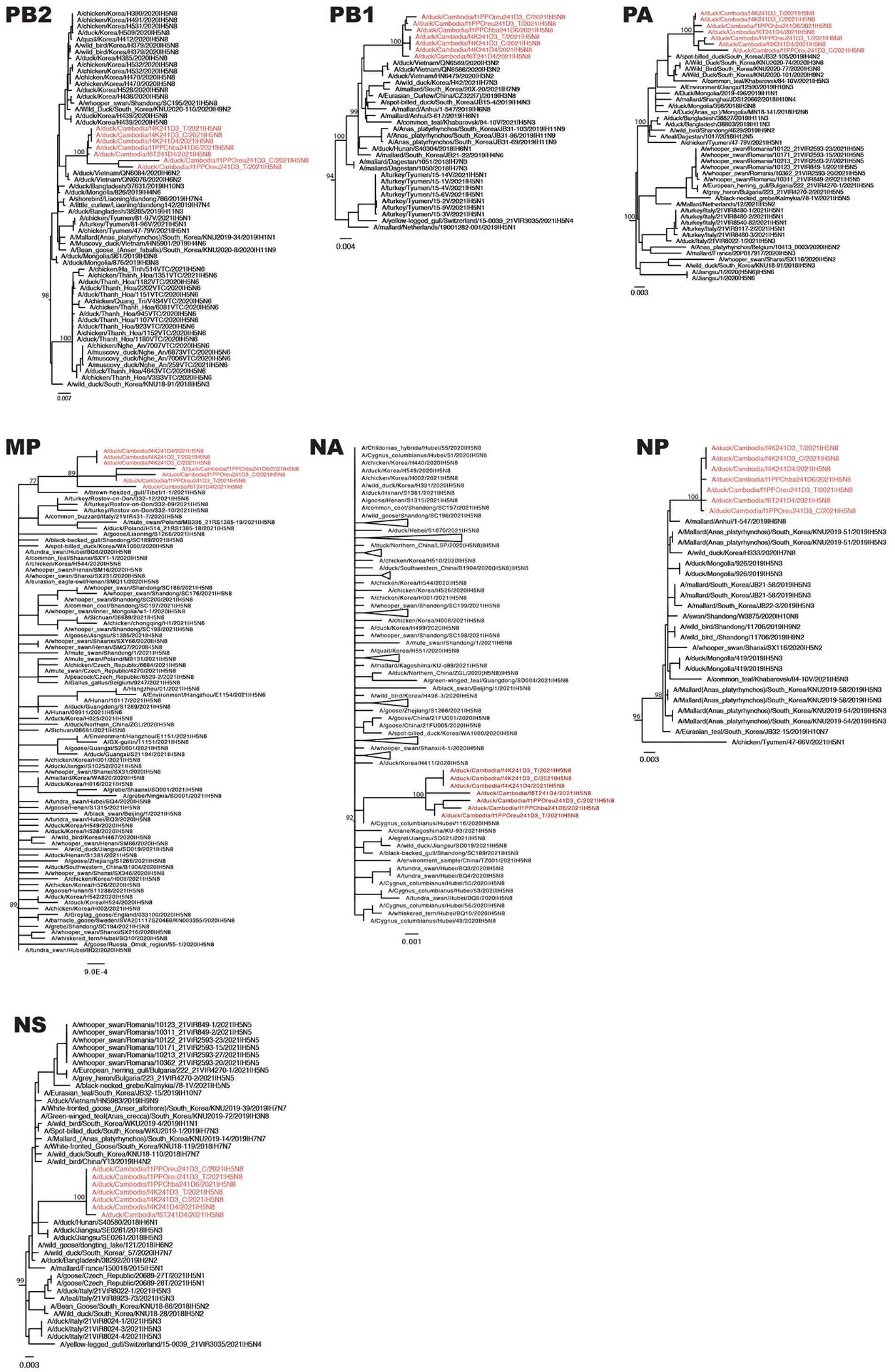

Appendix Figure 1. Phylogenetic analysis of different genes of avian influenza A(H5N8) viruses detected in Cambodia. Phylogenies were constructed using the maximum-likelihood method. Recent clade 2.3.4.4b isolates from Cambodia are in red font. Scale bars indicate nucleotide substitutions per site. MP, matrix protein; NA, neuraminidase; NP, nucleoprotein; NS, nonstructural; PA, polymerase acidic; PB1, polymerase basic 1; PB2, polymerase basic 2.

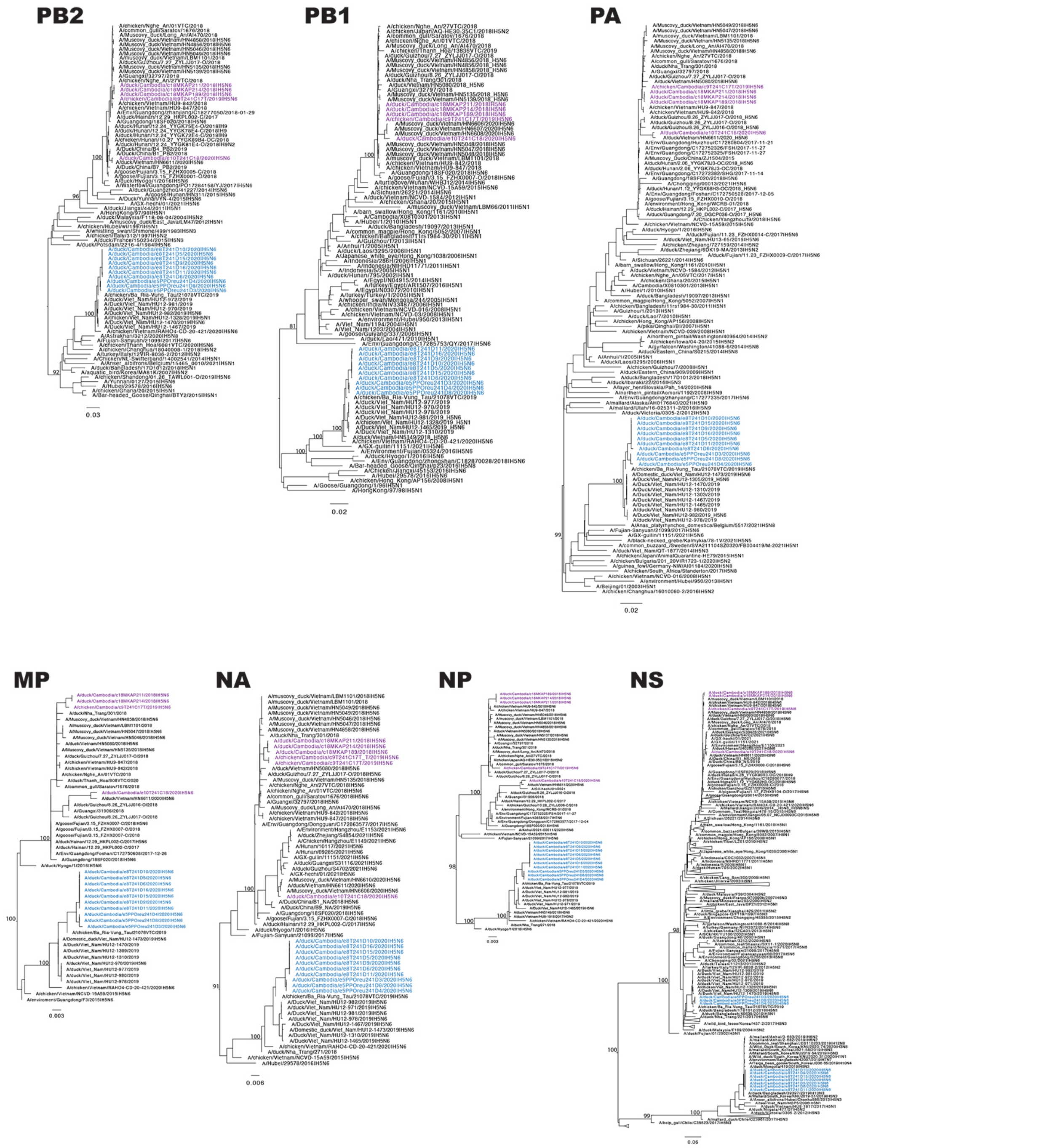

Appendix Figure 2. Phylogenetic analysis of different genes of avian influenza A(H5N6) viruses detected in Cambodia. Phylogenies were constructed using the maximum-likelihood method. Recent clade 2.3.4.4g (purple font) and 2.3.4.4h (blue font) isolates from Cambodia are shown. Scale bars indicate nucleotide substitutions per site. MP, matrix protein; NA, neuraminidase; NP, nucleoprotein; NS, nonstructural; PA, polymerase acidic; PB1, polymerase basic 1; PB2, polymerase basic 2.

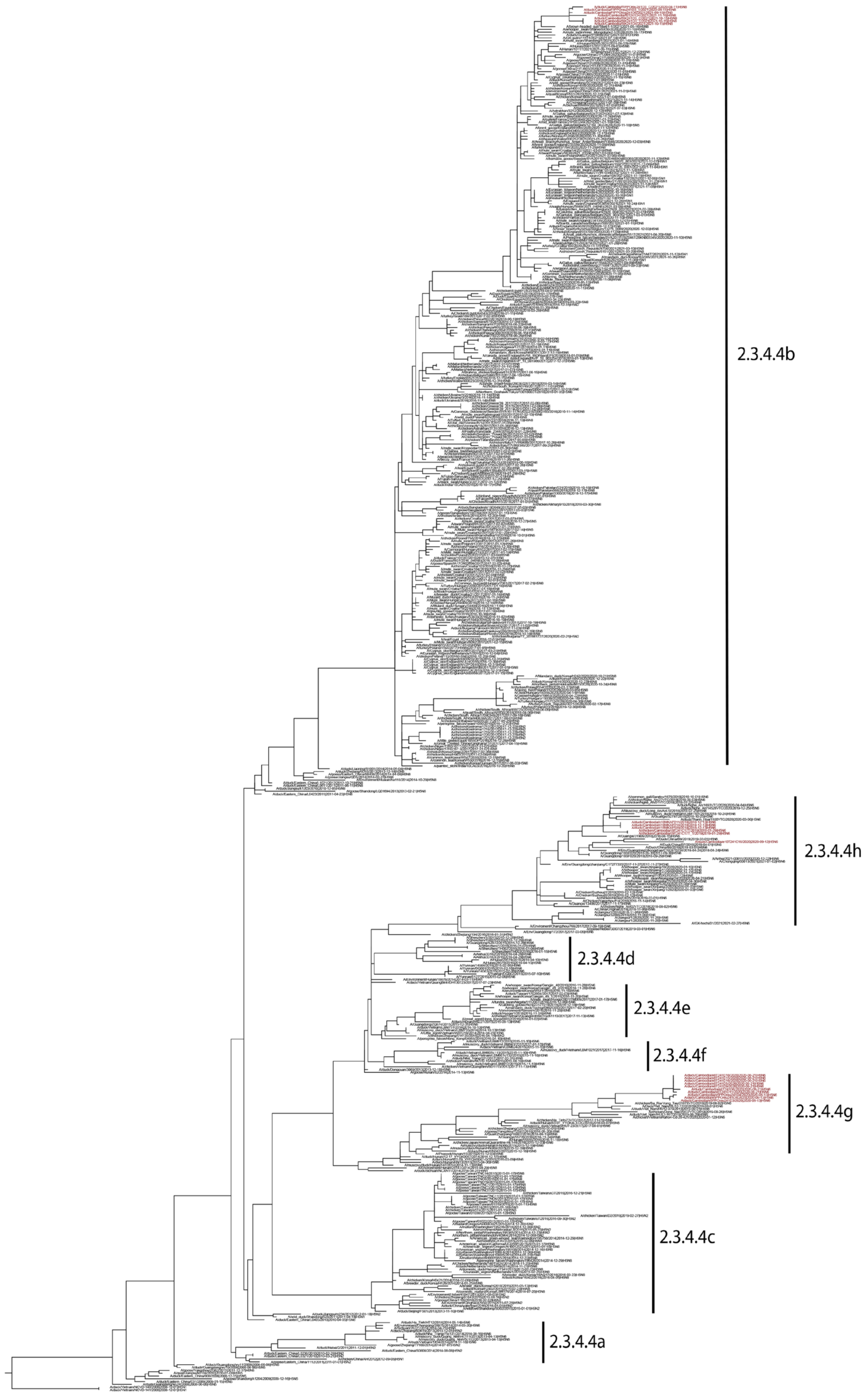

Appendix Figure 3. Phylogenetic analysis of the hemagglutinin gene of clade 2.3.4.4 viruses generated using the maximum-likelihood method. Recent isolates from Cambodia are in red font. Scale bar indicates nucleotide substitutions per site.

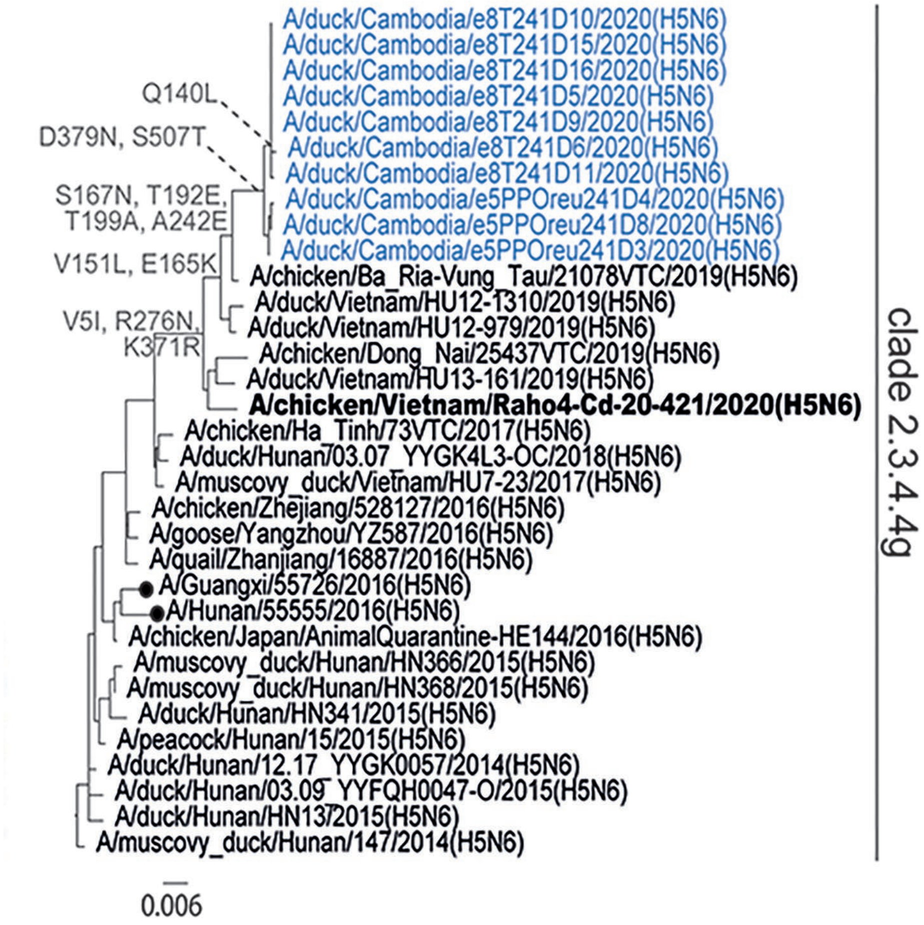

Appendix Figure 4. Phylogenetic analysis of hemagglutinin genes from avian influenza subtype H5N6 in study of clade 2.3.4.4 avian influenza viruses detected in Cambodia. Dendograms were constructed using maximum-likelihood phylogeny and show phylogeny of avian influenza A(H5N6) clade 2.3.4.4g isolates from Cambodia. Recent isolates from Cambodia are in blue font and amino acid mutations are indicated at select nodes. Candidate vaccine viruses used as reference viruses are in bold font. Closed circles indicate cases of human infection with avian H5N6 clade 2.3.4.4g viruses. Scale bar indicates nucleotide substitutions per site.

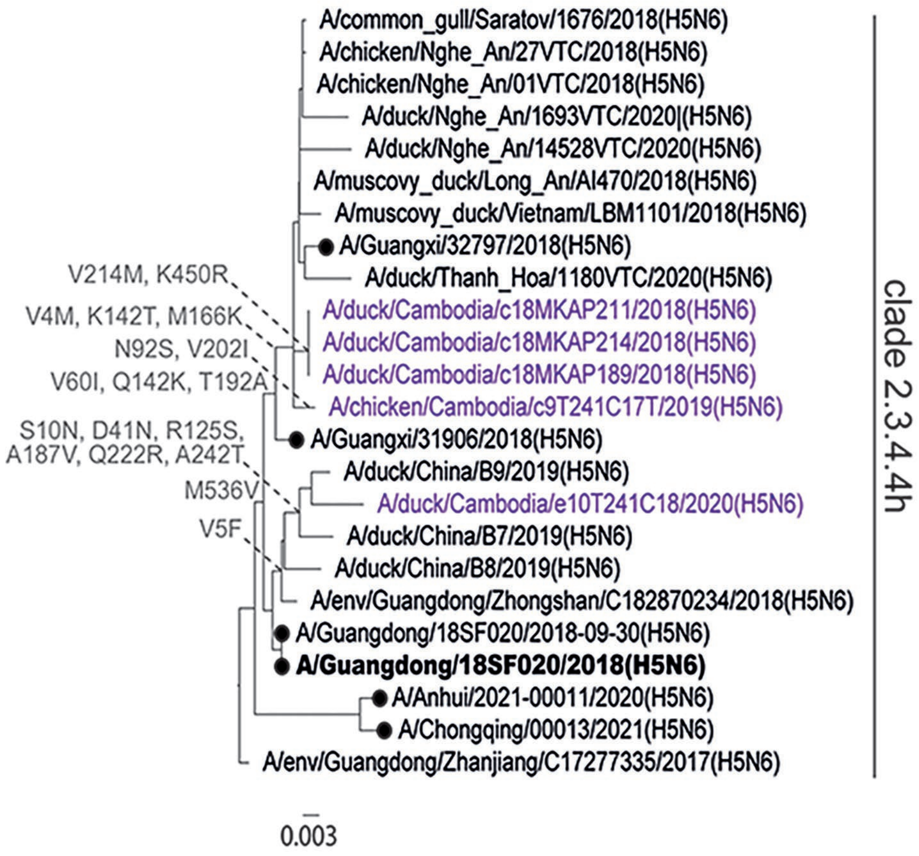

Appendix Figure 5. Phylogenetic analysis of hemagglutinin genes from avian influenza subtype H5N6 in study of clade 2.3.4.4 avian influenza viruses detected in Cambodia. Dendograms were constructed using maximum-likelihood phylogeny and show phylogeny of avian influenza A(H5N6) clade 2.3.4.4h isolates from Cambodia. Recent isolates from Cambodia are in purple font and amino acid mutations are indicated at select nodes. Candidate vaccine viruses used as reference viruses are in bold font. Closed circles indicate cases of human infection with avian H5N6 clade 2.3.4.4h viruses. Scale bar indicates nucleotide substitutions per site.
